# A Practical Diagnostic Approach to Pediatric Episodic Vestibular Syndrome

**DOI:** 10.3390/children13050583

**Published:** 2026-04-22

**Authors:** Mar Rey-Berenguel, Juan Manuel Espinosa-Sanchez

**Affiliations:** 1 Division of Otoneurology, Department of Otolaryngology, Hospital Universitario Virgen de las Nieves, 18014 Granada, Spain; marreyber@correo.ugr.es; 2 Division of Otolaryngology, Department of Surgery, University of Granada, 18071 Granada, Spain; 3 Otology and Neurotology Group CTS495, Instituto de Investigación Biosanitaria ibs.GRANADA, 18012 Granada, Spain; 4 Sensorineural Pathology Program, Centro de Investigación Biomédica en Red en Enfermedades Raras (CIBERER), 28029 Madrid, Spain

**Keywords:** pediatric episodic vestibular syndrome, recurrent vertigo of childhood, vestibular migraine of childhood, pediatric vertigo, phenotype-driven approach

## Abstract

**Highlights:**

**What are the main findings?**

- A phenotype-first framework can organize recurrent pediatric vestibular symptoms into clinically actionable phenotypes.
- Targeted history, focused bedside examination, and selective ancillary testing improve diagnostic orientation and help identify red flags.

**What is the implication of the main finding?**

- The approach may improve diagnostic accuracy, support more rational use of ancillary testing, and facilitate earlier recognition of clinically important disorders.
- Longitudinal reassessment is essential because phenotypes may evolve and require diagnostic reclassification over time.

**Abstract:**

Pediatric episodic vestibular syndrome (pEVS) is a frequent source of diagnostic uncertainty because recurrent vertigo, dizziness, and unsteadiness in children may arise from disorders with markedly different mechanisms, prognostic implications, and management pathways. Symptom descriptions are often imprecise, interictal examination may be normal, and similar recurrent attack patterns may reflect spontaneous, triggered, neurologic, autonomic, audiovestibular, or extravestibular conditions. This Perspective proposes a clinician-oriented, phenotype-first framework for the practical evaluation of pEVS, grounded in the International Classification of Vestibular Disorders and Bárány Society consensus criteria where available. The proposed approach begins with structured history taking and focused bedside examination, emphasizing the core symptom category, attack duration, trigger profile, and associated migraine, auditory, autonomic, and neurologic features. Recurrent attacks are then organized into clinically recognizable phenotypes, including spontaneous non-migraine and migraine-related presentations, auditory phenotypes, ultrabrief stereotyped attacks, trigger-related attacks, orthostatic/autonomic phenotypes, motion- or visually-triggered dizziness, episodic vertigo with transient neurologic signs, and anxiety-related presentations. Rather than providing an exhaustive etiologic review, this framework is intended to support bedside classification, guide selective ancillary testing, and facilitate longitudinal reassessment, as diagnostic reclassification may occur over time. A phenotype-driven approach may improve diagnostic reasoning, support more rational use of ancillary testing, and facilitate earlier recognition of both common and less frequent but clinically important disorders.

## 1. Introduction

Vertigo, dizziness, and unsteadiness are increasingly recognized across childhood and adolescence; population-based studies suggest a 5.3–5.6% prevalence of dizziness and imbalance in individuals aged 3–17 years [1,2]. In children and adolescents, pediatric episodic vestibular syndrome (pEVS) is a major diagnostic challenge because episodic vestibular symptoms are often described imprecisely, interictal examination may be normal, and similar recurrent attack patterns can arise from disorders with markedly different mechanisms and management.

Under the International Classification of Vestibular Disorders (ICVD) symptom taxonomy, vertigo denotes a false sensation of self-motion or a distorted sensation of self-motion during otherwise normal head movement, dizziness refers to disturbed or impaired spatial orientation without a false sense of motion, and unsteadiness describes balance symptoms occurring while upright [3]. At the syndromic level, the ICVD proposes three high-level vestibular syndromes that support pathway-based clinical reasoning: acute vestibular syndrome, episodic vestibular syndrome, and chronic vestibular syndrome [4]. pEVS encompasses recurrent attacks of vertigo, dizziness, and/or unsteadiness lasting from seconds to hours and separated by symptom-free or near-symptom-free intervals.

In this context, a phenotype-first framework may help clinicians translate imprecise symptom descriptions and overlapping attack patterns into a more structured differential diagnosis, support more selective ancillary testing, and facilitate longitudinal reassessment when the initial phenotype is only partially expressed. This need is particularly relevant in pediatric practice, where the application of existing diagnostic classifications is often complicated by age-dependent symptom vocabulary, limited symptom recall, and frequently normal interictal examination, in addition to the etiologic heterogeneity of pediatric dizziness itself. Previous studies have underscored these practical limitations, including variability in etiologic classification and the need for more structured clinical algorithms [5,6]. Rather than reviewing individual disorders in isolation, this Perspective provides an expert, clinician-oriented synthesis intended to structure the initial diagnostic reasoning in children presenting with pEVS. It is not intended as a formal guideline or a systematic review, but as a pragmatic, phenotype-first framework anchored in attack duration, triggers, and associated features, and aligned, where available, with Bárány Society consensus criteria [7].

## 2. Diagnostic Approach to Pediatric Episodic Vestibular Syndrome

The diagnostic approach to pEVS differs from that in adults because history taking and bedside evaluation require adaptation to age, symptom vocabulary, and cooperation, while the spectrum and relative frequency of the underlying disorders are also different. Whereas benign paroxysmal positional vertigo (BPPV) is the most common cause of adult vertigo, pEVS is dominated by recurrent vertigo of childhood (RVC) and vestibular migraine of childhood (VMC), while several less frequent but clinically important neurologic, autonomic, audiovestibular, and extravestibular disorders remain part of the differential diagnosis.

A structured history remains the cornerstone of pEVS evaluation. As a first step, the clinician should phenotype the complaint by clarifying (i) the core symptom category (vertigo vs. dizziness vs. unsteadiness), (ii) typical attack duration and temporal pattern, (iii) trigger profile, and (iv) key associated features. The trigger profile should be characterized carefully, including whether attacks are spontaneous or reproducibly triggered by head position change, arising or upright posture, active or passive motion, visually complex environments, exertion, sound, pressure changes, or Valsalva-like maneuvers. Key associated features include migraine symptoms, auditory symptoms, autonomic features, transient neurologic signs, and altered awareness. This history-based phenotyping establishes the initial clinical framework to be refined at bedside examination.

Subsequently, a focused bedside examination should confirm or challenge the suspected phenotype and identify central or otologic red flags. Depending on the clinical scenario, bedside assessment should include otoscopy; ocular-motor examination (alignment and vergence, saccades, smooth pursuit, spontaneous and gaze-evoked nystagmus, and visual vestibulo-ocular reflex suppression); positional testing (Dix-Hallpike maneuvers, supine roll test, and midline head-hanging position); selected dynamic vestibular bedside tests (e.g., head-impulse testing, post-rotatory nystagmus, and, in selected cases, skull vibration-induced nystagmus); and postural/cerebellar assessment (Romberg, Fukuda stepping, single-leg stance, past-pointing, finger-to-nose and finger-to-finger testing, rapid alternating movements, and gait observation). Head-shaking testing is generally better tolerated in adolescents than in younger children. Together, structured history and focused bedside examination provide the main basis for initial phenotypic classification and for deciding whether ancillary testing is needed.

Because some children with apparent pEVS may harbor central or other serious pathology, a small number of red flags should lower the threshold for urgent neuroimaging or expedited neurologic evaluation, as summarized in Table 1.

**Figure 1:**
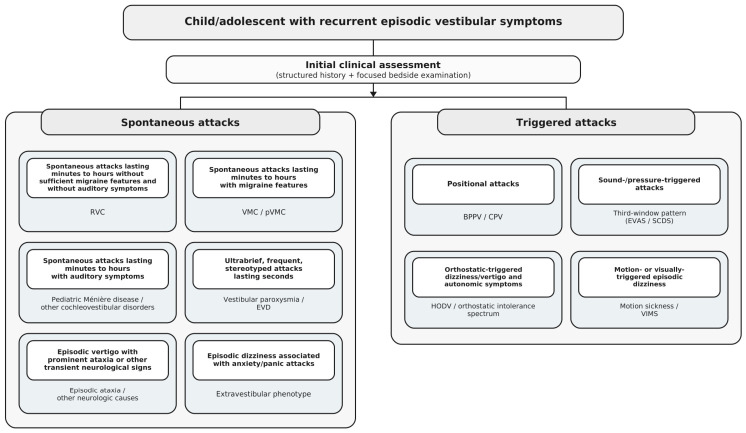
Phenotype-first diagnostic workflow for pediatric episodic vestibular syndrome. The figure outlines a phenotype-first approach integrating structured history-based phenotyping, focused bedside examination, and the main clinical branches of the differential diagnosis. Phenotypes are pragmatic entry points rather than final diagnoses. Abbreviations: RVC, recurrent vertigo of childhood; VMC, vestibular migraine of childhood; pVMC, probable vestibular migraine of childhood; BPPV, benign paroxysmal positional vertigo; CPV, central positional vertigo; EVD, epileptic vertigo/dizziness; EVAS, enlarged vestibular aqueduct syndrome; SCDS, superior canal dehiscence syndrome; HODV, hemodynamic orthostatic dizziness/vertigo; VIMS, visually induced motion sickness.

Figure 1 summarizes this phenotype-first diagnostic workflow for pEVS, integrating history-based phenotyping, focused bedside examination, and the main clinical branches that structure the differential diagnosis.

## 3. Operationalizing a Phenotype-Driven Approach

Based on the initial clinical assessment, recurrent attacks can be provisionally grouped into a limited number of clinically recognizable phenotypes. These are not final diagnoses, but pragmatic entry points for organizing the differential diagnosis of pEVS. In children and adolescents, overlap between phenotypes is common, and longitudinal follow-up may lead to diagnostic reclassification over time. In practice, at the RVC/pVMC/VMC interface, children who fulfill the required vestibular and duration criteria but present only one of the two migraine-related elements are better classified as pVMC than as RVC; a genuinely provisional classification is mainly reserved for cases in which symptom description is limited, key migraine features cannot be assessed reliably, or the criteria are not yet fully met and longitudinal follow-up is needed [7,8,9]. Accordingly, ancillary testing in pEVS should remain selective and phenotype-oriented rather than routine; its role is to clarify specific diagnostic questions raised by the history and bedside examination, as summarized in Table 2. The spectrum discussed below ranges from the most prevalent spontaneous pediatric syndromes to less frequent but clinically important triggered, hemodynamic, neurologic, and extravestibular patterns, encompassing not only primary vestibular disorders but also other recurrent presentations that may clinically manifest as pEVS. The following sections develop these phenotypes in a pragmatic clinical sequence, moving from the most common spontaneous pediatric syndromes to less frequent but clinically important triggered, auditory, hemodynamic, neurologic, and extravestibular presentations.

## 4. Phenotype-Driven Differential Diagnosis

### 4.1. Spontaneous Attacks Lasting Minutes to Hours Without Sufficient Migraine Features and Without Auditory Symptoms

When children present with recurrent spontaneous vestibular attacks lasting minutes to hours, without a migraine burden sufficient to support VMC or probable VMC (pVMC), and without dominant auditory symptoms, RVC becomes the leading diagnostic consideration [7]. In this practical framework, “without sufficient migraine features” should be understood as the absence of both a current or past history of migraine and the attack-related migraine feature burden required for VMC (Table 3); if only one of these elements is present, pVMC rather than RVC should be considered [7]. This distinction may be particularly difficult in younger children, in whom symptom description and recall are often limited by age. Likewise, “without auditory symptoms” should be understood as a practical phenotypic distinction rather than an absolute exclusion: persistent, fluctuating, or diagnostically prominent auditory symptoms should redirect the differential diagnosis toward audiovestibular disorders, including Meniere disease, whereas transient or nonspecific auditory complaints do not by themselves exclude RVC [10,11,12].

Within the 2021 Bárány Society/International Headache Society (IHS) framework, RVC, pVMC, and VMC are best regarded as part of a pediatric spectrum ranging from RVC, in which migraine may be absent, to VMC, in which migraine is definite, with pVMC occupying an intermediate position; however, this should not be interpreted as implying inevitable stepwise progression in every child [7].

#### Recurrent Vertigo of Childhood

According to the 2021 Bárány/IHS classification, RVC replaced the older International Classification of Headache Disorders (ICHD) term benign paroxysmal vertigo (BPV; termed benign paroxysmal vertigo of childhood, BPVC, in ICHD-2). Despite differences in formal diagnostic criteria, historical BPV/BPVC cohorts remain clinically closest to the contemporary RVC category.

Age at onset typically falls within childhood, although reported cohorts differ according to referral setting and case definition. Historical BPV/BPVC series often described onset before 4 years of age, whereas more recent tertiary-clinic RVC cohorts report symptom onset later in childhood, frequently around the early school years [13,14]. Symptom descriptions vary with age and vocabulary, but attacks are most commonly described as spinning or swaying vertigo; some children report dizziness or unsteadiness instead [11,14]. Most attacks are brief, often in the 1–5 min range, although longer episodes within the Bárány range may occur, and apparently prolonged attacks may sometimes reflect clustering of short recurrent spells rather than one continuous episode [7,11,14]. Common accompanying symptoms include nausea, vomiting, unstable gait or unsteadiness, fearful behavior, and occasionally non-migrainous headache [13,14].

Interictal neurologic and neuro-otologic examination is usually normal [14]. Mild ocular-motor abnormalities may occasionally be observed, including saccadic smooth pursuit, impaired fixation suppression, or central positional nystagmus, but these findings are nonspecific and do not reliably distinguish RVC from closely related VMC/pVMC phenotypes [10,14]. Vestibular testing may also reveal abnormalities on video head impulse testing (vHIT), caloric testing, or vestibular evoked myogenic potentials (VEMP) in some patients. Current evidence, however, does not support these tests as reliable tools for distinguishing RVC from pVMC or VMC [12,15].

The natural history is generally favorable, with many children showing remission or a clear reduction in attack frequency over time [14]. Nevertheless, some patients later develop migraine or are reclassified as pVMC or VMC, underscoring the importance of longitudinal follow-up [8,9]. Management is mainly conservative and preventive, emphasizing education, reassurance, hydration, sleep regularity, regular meals, physical activity, and follow-up [14].

### 4.2. Spontaneous Attacks Lasting Minutes to Hours with Migraine Features

When children present with recurrent spontaneous vestibular attacks lasting minutes to hours, and the overall migraine burden supports a diagnosis within the pediatric vestibular migraine spectrum, vestibular migraine of childhood (VMC) or probable vestibular migraine of childhood (pVMC) becomes the leading diagnostic consideration [7].

#### Vestibular Migraine of Childhood and Probable Vestibular Migraine of Childhood

The qualifying vestibular symptoms for VMC/pVMC are more specific than the broader symptom vocabulary used by children and families. According to the Bárány criteria, episodes may consist of spontaneous vertigo, positional vertigo, visually induced vertigo, head motion-induced vertigo, or head motion-induced dizziness with nausea [7,16]. Other complaints such as unsteadiness, motion sickness, or nonspecific dizziness may accompany attacks and still provide useful diagnostic context, but they do not by themselves satisfy the vestibular symptom criterion [7,16]. Migraine headache does not need to accompany every attack; photophobia and phonophobia, or visual aura, may fulfill the migraine-feature criterion in its place [7,17].

In children and adolescents, migraine headache is typically frontotemporal and more often bilateral than in adults, whereas unilateral pain tends to emerge later, particularly during adolescence. Occipital headache is uncommon and should prompt careful assessment for red flags; when warning features are present, neuroimaging should be obtained to exclude structural pathology. Many children with VMC/pVMC also experience nausea or vomiting during attacks, post-episode tiredness or sleepiness, and a positive family history of migraine. Transient auditory symptoms may occur in a subset, particularly in children fulfilling VMC criteria, without necessarily implying a primary audiovestibular disorder such as Meniere disease [10,11,12,18].

Interictal neurologic and neuro-otologic examination is often normal, although mild ocular-motor abnormalities including saccadic smooth pursuit or central positional nystagmus may occasionally be found [10]. These findings are nonspecific and should not be overinterpreted. Insufficient convergence and refractive error should also be excluded [19].

Audiometry is appropriate when auditory complaints are reported. Vestibular function tests such as vHIT, caloric testing, or VEMP may document vestibular dysfunction in individual cases, but the distinction between pVMC/VMC and RVC still relies primarily on clinical phenotype rather than on these ancillary tests [12,15].

VMC/pVMC may overlap with or be clinically confused with other pediatric vestibular syndromes. Reproducible positional provocation should redirect attention toward positional phenotypes, whereas persistent daily symptoms exacerbated by upright posture, motion, and visually complex environments should shift the differential toward persistent postural-perceptual dizziness (PPPD) rather than an ongoing episodic migraine phenotype [18,20].

First-line care is largely extrapolated from pediatric migraine practice and emphasizes education, sleep regularity, hydration, regular meals, physical activity, trigger control, and attention to comorbidities such as anxiety, motion sensitivity, and school impact [17,18,21]. When attack burden is substantial, migraine-oriented preventive treatment may be considered, but pediatric evidence specific to vestibular outcomes remains limited [17,21].

### 4.3. Spontaneous Attacks Lasting Minutes to Hours with Auditory Symptoms

Vertigo attacks accompanied by fluctuating auditory symptoms (hearing changes, tinnitus, or aural fullness) should raise suspicion for pediatric Meniere disease and other audiovestibular disorders, including enlarged vestibular aqueduct syndrome (EVAS) in the appropriate clinical context.

#### Pediatric Meniere Disease

Meniere disease (MD) is rare in childhood but remains clinically relevant because its episodic vertigo phenotype may overlap with VMC/pVMC and other recurrent vertigo disorders, while premature diagnostic anchoring on “migraine” may delay recognition of progressive cochlear involvement. In a large tertiary series, pediatric-onset MD (<15 years) accounted for approximately 2.3% of patients with MD, underscoring its rarity but also its practical importance in specialized settings [22].

Although MD is classically associated with fluctuating auditory symptoms, children may describe tinnitus, hearing fluctuations, or aural fullness less reliably than adults, and these symptoms may evolve gradually over time. Accordingly, longitudinal follow-up with serial audiometry is especially important in suspected pediatric MD [22]. In that series, audiometric abnormalities were the most frequent finding, followed by alterations in cervical vestibular evoked myogenic potentials (cVEMP), ocular vestibular evoked myogenic potentials (oVEMP), and caloric responses, suggesting a cochlea-to-saccule/utricle/canal pattern similar to that described in adult MD [22].

Pediatric MD should be suspected when recurrent vertigo coexists with objectively documented, fluctuating low-frequency sensorineural hearing loss, consistent with the Bárány Society criteria for definite/probable Menière’s disease [23]. By contrast, isolated transient tinnitus or aural fullness may also occur in VMC/pVMC and should not, by themselves, anchor the diagnosis of MD. The presence of migraine does not exclude MD, but progressive or objectively fluctuating sensorineural hearing loss warrants careful reassessment of the diagnosis. When hearing loss is unilateral or asymmetrically bilateral, brain MRI is often obtained to exclude alternative retrocochlear or central causes. EVAS should also be considered when episodic vertigo occurs in a child with early-onset, fluctuating, or progressive hearing loss, particularly when hearing or vestibular symptoms worsen after minor head trauma or barotrauma [24,25].

Management evidence in pediatric MD is limited and largely extrapolated from adult practice. Initial care usually emphasizes conservative measures, serial audiometric monitoring, and early attention to hearing rehabilitation; invasive therapies are reserved for exceptional refractory cases after multidisciplinary assessment.

### 4.4. Ultrabrief, Frequent, Stereotyped Attacks Lasting Seconds

Very brief (seconds), highly stereotyped attacks—often recurring many times per day—should raise suspicion for vestibular paroxysmia (VP). Epileptic vertigo/dizziness (EVD) is an important mimic, particularly when episodes are accompanied by altered awareness, cognitive change, or other ictal features [26,27,28].

#### 4.4.1. Vestibular Paroxysmia

Vestibular paroxysmia (VP) is an uncommon but clinically important cause of ultrabrief recurrent vestibular attacks in children and adolescents because it is potentially treatable and may be under-recognized [26,29].

Bárány Society criteria distinguish VP and probable VP (Table 4). Marked intra-individual stereotypy is highly suggestive, and some attacks may be triggered by certain head movements, although these do not follow the typical positional pattern of BPPV [26]. Episodes may also be accompanied by transient unsteadiness or auditory symptoms during attacks, such as unilateral tinnitus or hyperacusis [26].

VP is thought to result from ephaptic discharges in the proximal vestibulocochlear nerve, usually in the setting of neurovascular cross-compression [26]. In children, additional anatomical substrates should also be considered. A pediatric cohort highlighted a narrowed internal auditory canal as a distinct associated phenotype, particularly in adolescents and females, supporting the use of high-resolution MRI of the cerebellopontine angle and internal auditory canals when VP is suspected [30].

Interictal bedside examination is often normal, although hyperventilation-induced nystagmus may provide supportive evidence when present [26]. Audiometry is appropriate when auditory symptoms are reported. Ancillary testing may show delayed auditory conduction on auditory brainstem response (ABR), whereas vestibular tests such as vHIT, caloric testing, or VEMP are often normal or only mildly abnormal [31].

High-resolution MRI of the cerebellopontine angle is primarily used to exclude secondary causes and to characterize relevant anatomy; importantly, neurovascular contact or vascular loops are not diagnostic in isolation because similar findings are common in asymptomatic individuals [26,32,33]. When the phenotype is strongly suggestive of VP and alternative diagnoses have been reasonably excluded, a trial of carbamazepine or oxcarbazepine may be both therapeutically useful and diagnostically supportive; pediatric reports describe oxcarbazepine as well tolerated, and adult experience suggests a more favorable tolerability and interaction profile than carbamazepine, although pediatric evidence remains limited [29].

#### 4.4.2. Vestibular Epilepsy/Epileptic Vertigo and Dizziness

EVD is a rare differential diagnosis of ultrabrief recurrent vestibular episodes [27,28]. Isolated EVD appears to be uncommon, and when duration is reported, very brief episodes (≤30 s) are more typical of isolated than non-isolated presentations [27]. Any associated alteration of awareness, cognitive change, or other clearly ictal features should heighten suspicion for EVD, especially in children [28].

Diagnostic work-up should include electroencephalography (EEG) and brain MRI, while vestibular testing may help exclude peripheral mimics [28]. A therapeutic response to anti-seizure medication does not by itself prove an epileptic origin, because VP may also respond to sodium-channel blockers [27,29].

### 4.5. Trigger-Related Attacks

Triggered phenotypes comprise recurrent vestibular episodes that are consistently linked to specific provoking circumstances rather than occurring truly spontaneously. In pEVS, the most clinically relevant triggered phenotypes include positional attacks, sound-/pressure-triggered attacks, orthostatic-triggered dizziness/vertigo with autonomic symptoms, and motion- or visually-triggered episodic dizziness. Distinguishing among these patterns helps refine the differential diagnosis and guides the focused examination and selective ancillary work-up.

#### 4.5.1. Positional Attacks

Benign paroxysmal positional vertigo in children

BPPV is a paroxysmal, position-triggered vestibular disorder caused by displaced otoconia within a semicircular canal or, less commonly, adherent to the cupula. Formal diagnostic criteria for BPPV have been established by the Bárány Society and provide the reference framework for diagnosis once the appropriate positional nystagmus pattern is elicited [34]. In children, it has traditionally been considered uncommon, but contemporary pediatric series and recent evidence syntheses suggest that it is often under-recognized when a standardized positional examination is not performed [35,36]. BPPV is clinically important because it is treatable, may occur after concussion or head trauma, and may coexist with VMC/pVMC [18,37,38].

A targeted history should probe explicitly for very brief episodes—typically seconds and occasionally up to about 1 min—and for reproducible positional triggers such as lying down, rolling in bed, turning the head in bed, looking up, or bending over. Symptoms may be better tolerated than in adults, and younger children may not describe classic spinning vertigo; caregivers may instead report brief fear, pallor, nausea or vomiting, clinging behavior, transient unsteadiness, or avoidance of lying down or turning in bed [35,39]. Auditory symptoms are not typical of isolated BPPV. When positional episodes are accompanied by fluctuating hearing loss, tinnitus, or aural fullness, alternative audiovestibular or pressure-related disorders should be considered, including EVAS in the appropriate clinical context [35].

Posterior-canal BPPV typically produces a transient torsional upbeating nystagmus on Dix–Hallpike maneuver. Horizontal-canal BPPV produces horizontal nystagmus on the supine roll test, usually geotropic in canalithiasis and apogeotropic in cupulolithiasis. Anterior-canal BPPV may produce downbeating nystagmus on a midline head-hang position, usually with a torsional component. Compared with adult practice, pediatric BPPV may show a relatively higher proportion of horizontal, superior, multicanal, or recurrent forms [35,37,40].

A practical first step is to distinguish genuinely positionally triggered attacks, which favor BPPV, from symptoms that are merely positionally exacerbated [41,42]. Within pEVS, the most consequential mimic is central positional vertigo (CPV), expressed on examination as central positional nystagmus (CPN). A common diagnostic error is positional bias: over-inferring a benign peripheral cause whenever dizziness is reported with positional features.

CPV should be suspected when positional testing elicits CPN with a canal-incongruent vector, a predominantly vertical component, poor fatigability, persistent nystagmus while the position is maintained, lack of response to appropriate repositioning maneuvers, or additional central ocular-motor or neurologic signs (Table 5). In such cases, posterior fossa-focused brain MRI should be strongly considered [41,42].

#### 4.5.2. Sound-/Pressure-Triggered Attacks

This phenotype comprises recurrent episodes of vertigo, dizziness, oscillopsia, or unsteadiness that are reproducibly triggered by sound, pressure changes, or Valsalva-like maneuvers rather than being truly spontaneous. Relevant triggers include loud sounds, coughing, sneezing, nose blowing, straining, exertion, barometric pressure changes, and in some children, symptom worsening after minor head trauma or barotrauma. Within pEVS, this pattern should raise suspicion for third-window disorders, particularly enlarged vestibular aqueduct syndrome (EVAS) and superior canal dehiscence syndrome (SCDS), while also keeping perilymphatic fistula in the differential diagnosis [3,43,44].

Enlarged Vestibular Aqueduct Syndrome

EVAS should be suspected when recurrent vestibular attacks occur in a child with delayed-onset, fluctuating, progressive, or sudden sensorineural hearing loss, particularly when hearing or vestibular symptoms worsen after head trauma, barotrauma, or viral illness. EVAS is the most common structural inner-ear anomaly identified on imaging in children with congenital hearing loss. It is frequently associated with SLC26A4 variants, particularly in DFNB4 and Pendred syndrome, and may coexist with incomplete partition type II (Mondini malformation) and/or vestibular and semicircular canal anomalies [24,25,45]. Reported vestibular manifestations include rotatory vertigo, clumsiness or unsteadiness, episodes of head tilt with vomiting in early childhood, and delayed independent walking; a greater vestibular symptom burden has been associated with bilateral radiologic EVA and with a history of head injury [46]. Audiologically, EVAS may produce pseudo-conductive or mixed hearing loss despite normal middle-ear status, and VEMP testing supports a third-window mechanism, with low-threshold responses being particularly characteristic [24,47]. Clinical suspicion often arises from the chronology of hearing loss, the association with precipitating events, and confirmatory temporal bone imaging [24].

Superior Canal Dehiscence Syndrome

Superior canal dehiscence syndrome (SCDS) should be considered in children and adolescents with recurrent vestibular symptoms, especially when episodes are reproducibly triggered by sound, pressure changes, or Valsalva-like maneuvers. In pediatric practice, however, these features may be incompletely expressed or difficult to recognize, as younger children may describe only vague dizziness, unsteadiness, or discomfort rather than typical third-window symptoms. Auditory manifestations should therefore be actively explored, particularly bone-conduction hyperacusis, autophony, and other abnormally loud perceptions of internal body sounds (e.g., eye movements or blinking, jaw or neck crepitus, borborygmi, and footfalls), as well as pulsatile tinnitus. Audiological suspicion is reinforced when testing shows a low-frequency air-bone gap, often within a conductive or mixed hearing-loss pattern and sometimes accompanied by negative low-frequency bone-conduction thresholds, despite normal otoscopy and largely normal middle-ear function, usually with normal tympanometry and often intact otoacoustic emissions; this pattern should raise suspicion for a third-window lesion rather than primary middle-ear disease [48]. Further physiological support is provided by enhanced VEMP responses, classically low cVEMP thresholds and/or high oVEMP amplitudes [43,48]. Nevertheless, high-resolution temporal bone CT with multiplanar reconstruction is required to demonstrate the bony defect, but imaging alone does not establish SCDS, because an apparent dehiscence may also be seen in children who do not have the full syndrome [43]. The diagnosis should therefore rest on concordance between compatible symptoms, supportive physiological findings, and radiologic evidence, while excluding more likely alternative explanations. Consensus diagnostic criteria are summarized in Table 6 [43].

#### 4.5.3. Orthostatic-Triggered Dizziness/Vertigo and Autonomic Symptoms

In children and adolescents, dizziness or vertigo reproducibly triggered by standing up or during upright posture—especially when accompanied by palpitations, presyncope, nausea, or visual blurring—strongly suggests an orthostatic mechanism and warrants evaluation for orthostatic intolerance syndromes, including postural orthostatic tachycardia syndrome (POTS) [49,50,51]. In this setting, the Bárány Society/ICVD consensus criteria for hemodynamic orthostatic dizziness/vertigo (HODV) provide a useful diagnostic reference (Table 7) [49].

In pediatric practice, the broader framework of orthostatic intolerance is often more clinically useful, since children and adolescents commonly present with orthostatic symptom clusters including lightheadedness, visual blurring, weakness, fatigue, nausea, palpitations, or near-syncope, with or without formal tilt-table testing [50,51]. A key practical distinction is between symptoms triggered by standing up or sustained upright posture, which support an orthostatic mechanism, and symptoms triggered by changes in head position relative to gravity, which favor a positional rather than hemodynamic cause [49].

Orthostatic symptoms are typically context-dependent, arise within minutes of standing or during continued upright posture, and improve with sitting or recumbency. Younger patients may describe “seeing black”, weakness, tremulousness, cognitive clouding, or impending faintness rather than true rotational vertigo. Common precipitants include heat, dehydration, prolonged standing, post-viral states, and exertion [18,49]. Because these episodes may coexist with VMC/pVMC or PPPD, orthostatic assessment should form part of the practical evaluation of pEVS. Syncopal events or presyncope require careful characterization of prodrome, duration, recovery, and injury risk.

Initial evaluation should include orthostatic heart rate and blood pressure measurements after at least 5 min supine and again at 1 and 3 min after standing; when feasible, assessment may be extended to 10 min while documenting symptom reproduction [49,50]. In adolescents, POTS is defined by chronic orthostatic intolerance symptoms with an excessive heart rate increment on standing in the absence of orthostatic hypotension, using pediatric-specific rather than adult thresholds [18,50]. When red flags are present—such as exertional syncope, chest pain, family history of sudden cardiac death, abnormal cardiac examination, or abnormal ECG—cardiology evaluation is mandatory [50,51].

First-line management is primarily non-pharmacologic and centers on adequate hydration, increased dietary salt when not contraindicated, avoidance of prolonged standing and heat, physical counter-maneuvers, compression garments, and structured aerobic and resistance conditioning [50,51]. Education is also essential, including recognition of prodromal symptoms and strategies to reduce injury risk. Pharmacologic treatment is reserved for refractory cases and should be guided by pediatric cardiology or autonomic specialists [50]. Although many adolescents improve over time with lifestyle measures and reconditioning, persistent symptoms may still affect school attendance, physical activity, and quality of life [50].

#### 4.5.4. Motion- or Visually-Triggered Episodic Dizziness

A consistent and reproducible relationship between episodic dizziness and passive physical motion (e.g., car travel, boats, or amusement rides) and/or visual motion in visually complex environments (e.g., optokinetic scenes, simulators, or virtual/augmented reality) supports a motion- or visually-triggered phenotype in children and adolescents. Within pEVS, this pattern may occur in isolation, but it often coexists with migraine susceptibility and may therefore help refine phenotypic classification [52,53,54].

For practical purposes, this phenotype can be approached using the Bárány Society classification for motion sickness (MS), visually induced motion sickness (VIMS), and their disorder forms (Table 8) [52].

Susceptibility appears to increase from infancy to a prepubertal peak and to decline thereafter, although the precise age distribution varies across studies [53]. Severe motion sickness may nevertheless occur even in infants and toddlers, and in pediatric dizziness cohorts recurrent or marked motion sickness is frequently associated with migraine liability [54].

The diagnosis is primarily clinical and depends on a clear temporal relationship between symptom onset and exposure to physical or visual motion. The main practical distinction is whether symptoms are truly trigger-dependent or instead represent spontaneous episodic dizziness with secondary motion intolerance. MS and VIMS are typically reproducible, build during continued exposure, and improve after the provoking stimulus ends. By contrast, spontaneous episodic syndromes within pEVS—particularly VMC/pVMC and RVC—may include motion or visual intolerance, but their defining attacks are not contingent on a specific external motion or visual trigger [52,53]. When symptoms arise predominantly after travel has ended rather than during exposure, alternative diagnoses such as mal de débarquement syndrome should be considered [52].

Interictal symptoms also deserve attention. Persistent unsteadiness, visual dependence, avoidance of visually busy environments, or marked anticipatory distress may suggest incomplete recovery, functional amplification, or a secondary persistent overlay rather than a purely episodic trigger-bound disorder. In such cases, PPPD-like perpetuation mechanisms and anxiety-related factors should be considered, particularly in adolescents with prolonged symptom burden [52].

Management is primarily non-pharmacologic and centers on trigger reduction, visual anchoring, optimization of seating and ventilation, and reinforcement of sleep and hydration measures. In selected patients, graded habituation may be helpful. Symptomatic medication can be considered in frequent or severe cases according to age and local practice, whereas vestibular rehabilitation may be useful in a subset of refractory patients, although supporting pediatric evidence remains limited [54].

### 4.6. Episodic Vertigo with Prominent Ataxia or Other Transient Neurologic Signs

In children and adolescents, recurrent vertigo or dizziness accompanied by transient gait ataxia, limb incoordination, dysarthria, or other focal neurologic signs should raise suspicion for episodic ataxia (EA) and related channelopathies, particularly when a suggestive family history or interictal ocular-motor abnormalities are present. Early recognition is important because EA is uncommon within pEVS, yet its paroxysmal presentation may overlap substantially with VMC and RVC, while the diagnostic work-up and treatment pathway are distinct and may require genetic confirmation and targeted pharmacologic treatment [55,56,57].

In pediatric cohorts, attacks typically last from minutes to hours and may be accompanied by prominent walking difficulties, marked unsteadiness, pallor, nausea, vomiting, and speech disturbance; physical exertion appears to be a particularly informative trigger favoring EA over VMC or RVC. Delayed motor and/or cognitive development and a positive family history are particularly suggestive when present [55]. Among genetically defined episodic ataxia syndromes, EA1 and EA2 are the most clinically relevant in pediatric practice. EA1, usually associated with KCNA1 variants, typically causes brief attacks lasting seconds to minutes and is classically associated with interictal myokymia. EA2, most often linked to CACNA1A variants, usually causes longer attacks lasting minutes to hours, often with interictal gaze-evoked nystagmus and frequent migraine comorbidity; most genetically defined EA2 cases begin before 20 years of age [56,57].

Interictal ocular-motor abnormalities are frequent and may provide particularly useful diagnostic clues when the attack history is ambiguous. These may include gaze-evoked nystagmus, downbeat nystagmus, impaired smooth pursuit, saccadic pursuit, or impaired fixation suppression, and in some patients mild persistent truncal ataxia may also be present [55]. A structured neurologic examination and systematic ocular-motor assessment are therefore essential in any child with recurrent vertigo and transient ataxia.

Because no formal pediatric consensus diagnostic criteria for episodic ataxia are currently available, the suggested clinical criteria summarized in Table 9 may be used as a pragmatic framework to identify children in whom genetic evaluation should be considered [55].

In children with moderate-to-high clinical suspicion of EA, the evaluation should usually extend beyond a routine vestibular work-up to include brain MRI to exclude structural pathology and genetic testing, ideally using next-generation sequencing panels, given the marked genetic heterogeneity and phenotypic overlap across episodic ataxia syndromes [55]. Cerebellar atrophy or other cerebellar abnormalities may be found in selected cases but are not required for diagnosis.

The main diagnostic pitfall is to over-attribute recurrent vertigo with pallor, nausea, or migraine features to RVC or to VMC/pVMC. In EA, the combination of exertional triggering, speech disturbance, gait ataxia, and reproducible interictal ocular-motor deficits warrants reconsideration of the diagnosis [55]. Conversely, not every child with mild interictal nystagmus has EA; medication effects, visual or refractive problems, and other central disorders, including central positional nystagmus when clinically appropriate, should also be considered in the differential diagnosis [55].

Treatment should be guided by pediatric neurology. Acetazolamide is commonly used as first-line prophylaxis, particularly in EA2. When it is ineffective or not tolerated, an aminopyridine such as 4-aminopyridine or its prolonged-release formulation (fampridine/dalfampridine) may be considered in selected cases under specialist supervision [55,58,59,60].

### 4.7. Episodic Dizziness Associated with Anxiety/Panic Attacks (Extravestibular Phenotype)

In children and adolescents, anxiety-related presentations—particularly panic attacks, with or without panic disorder—may present with recurrent episodes of dizziness and should be considered a distinct extravestibular phenotype within the differential diagnosis of pEVS. Episodes usually consist of dizziness, lightheadedness, subjective imbalance, unsteadiness, or non-spinning vertigo rather than true rotational vertigo, and are commonly accompanied by abrupt fear, autonomic arousal, hyperventilation, chest discomfort, tremor, paresthesias, derealization, and heightened bodily vigilance. Panic attacks are episodic phenomena rather than diagnoses in themselves and may occur in panic disorder or across other anxiety-related or psychiatric conditions. Because these manifestations may resemble neurologic or vestibular attacks, affected patients may initially present to pediatric, otolaryngology, neurology, or neuro-otology clinics with apparent episodic vestibular symptoms despite the absence of an identifiable primary vestibular disorder. Historical pediatric reports already recognized dizziness as a presenting symptom in panic disorder, often in the setting of otherwise unrevealing neurologic assessment [61].

This phenotype is clinically important because anxiety-related disorders account for a meaningful proportion of pediatric dizziness presentations, particularly in adolescents, and diagnostic overlap is common. Pediatric and neuro-otologic series suggest that anxiety-related and other extravestibular presentations are not uncommon, whereas more recent tertiary data indicate that anxiety disorders often coexist with, rather than exclude, other dizziness diagnoses [62,63,64]. Anxiety may amplify symptom perception, reinforce avoidance behavior, and increase overall functional burden even when a genuine vestibular disorder is also present.

This presentation should remain conceptually distinct from PPPD, which is a chronic functional vestibular disorder rather than an episodic one. By Bárány Society criteria, PPPD requires dizziness, unsteadiness, or non-spinning vertigo on most days for at least 3 months, typically worsened by upright posture, motion, and exposure to moving or visually complex environments [20]. The relationship between these two entities remains clinically important, because PPPD may be precipitated by psychological distress, including panic-like episodes, as well as by vestibular illness, and pediatric PPPD often coexists with VMC, BPPV, and anxiety [20,65].

In practice, the distinction between primary vestibular disorders and anxiety-driven episodic dizziness relies mainly on temporal pattern, trigger context, and associated symptoms. Primary vestibular disorders usually produce stereotyped attacks with phenotype-congruent vestibular triggers and accompanying migraine, auditory, neurologic, or orthostatic features, whereas anxiety-driven episodes are more often dominated by abrupt fear, hyperventilation, palpitations, tremor, derealization, anticipatory worry, and situational avoidance. PPPD should be suspected when symptoms are no longer confined to discrete attacks but are present on most days for at least 3 months and are consistently exacerbated by upright posture, motion, and visually complex environments. In diagnostically uncertain cases, a simple symptom diary may be useful to document episode frequency, duration, triggers, associated vestibular versus autonomic symptoms, and possible evolution from an episodic pattern to a persistent daily syndrome [20,65].

Accordingly, episodic dizziness associated with anxiety or panic attacks should be recognized as a separate extravestibular phenotype within pEVS, while acknowledging that it may coexist with genuine vestibular disease and that, in a subset of patients, a chronic functional dizziness syndrome such as PPPD may later emerge. Careful evaluation for concomitant vestibular, orthostatic, neurologic, cardiovascular, or metabolic causes remains essential whenever the history or examination suggests an alternative or additional explanation for the attacks.

## 5. Key Pitfalls in pEVS Classification

Several pitfalls complicate the classification of pEVS. One of the most common is the imprecise use of symptom terminology, particularly when non-vertiginous dizziness or unsteadiness is labeled as vertigo. Diagnostic imprecision also arises when orthostatic, anxiety-related, motion-triggered, positional, and spontaneous attacks are not clearly distinguished during history taking, or when associated auditory, neurologic, autonomic, or psychological features are not adequately integrated into the clinical interpretation.

Another frequent error is to over-rely on normal interictal vestibular testing, which does not exclude an episodic vestibular disorder. Equally problematic is the under-recognition of non-classical phenotypes, especially orthostatic and anxiety-related presentations, which may be overlooked when the evaluation is framed too narrowly around vestibular migraine or recurrent vertigo of childhood.

For example, recurrent dizziness triggered by standing should not be prematurely labeled as vestibular migraine when the history is more consistent with orthostatic intolerance, and positional symptoms should not be assumed to represent BPPV when the elicited nystagmus is persistent or canal-incongruent [41,42,49].

A further challenge is premature diagnostic closure. In many children, phenotypes evolve over time, and longitudinal follow-up is often necessary before the most appropriate classification becomes clear. This is particularly relevant along the spectrum from RVC to pVMC and VMC, but also in patients who later develop additional features pointing to an alternative or overlapping diagnosis.

Because phenotypic evolution and diagnostic reclassification may occur over time, follow-up is an integral part of pEVS evaluation rather than an optional add-on. In practical terms, children with a typical benign phenotype and no red flags may be reassessed clinically within 6–12 months, whereas earlier follow-up (e.g., within 3–6 months) is advisable when the phenotype is diagnostically uncertain, migraine features are incomplete or evolving, attack burden is high, or symptoms interfere substantially with daily activities. Repeat diagnostic work-up should be considered when new auditory symptoms emerge, the attack pattern changes substantially, interictal abnormalities appear, episodes become more prolonged or severe, or new focal neurologic, positional, or orthostatic red flags develop. In contrast, routine repetition of vestibular, imaging, or electrophysiologic studies is usually unnecessary in clinically stable children with an unchanged benign phenotype [7,8,9,14].

## 6. Conclusions

A syndromic, phenotype-driven approach offers a practical and clinically useful framework for the diagnosis of pEVS. In most children, the differential diagnosis is ultimately concentrated within a limited group of relatively common etiologies, whereas a smaller but important subset includes less frequent structural, neurologic, hemodynamic, psychological, anxiety-related, or systemic disorders. Careful history taking, targeted bedside examination, and selective ancillary testing remain the key elements for distinguishing among these phenotypes and guiding appropriate referral and management. Rather than relying on a single diagnosis-oriented algorithm, clinicians should approach pEVS as a structured phenotypic field in which trigger profile, attack semiology, associated features, and evolution over time together inform diagnostic probability. By organizing recurrent pediatric vestibular symptoms into pragmatic clinical phenotypes, this approach may improve diagnostic accuracy, support more rational use of ancillary testing, and facilitate earlier recognition of both common and less frequent but clinically important disorders.

## Figures and Tables

**Table 1:** Red flags in children with pEVS that should prompt urgent neuroimaging or expedited neurologic evaluation.

Domain	Red Flags
New focal or progressive neurologic signs	Persistent, progressive, or clearly new gait ataxia; cranial nerve palsy (e.g., diplopia, facial weakness); hemiparesis, dysarthria, language disturbance, or visual field deficit; altered consciousness; seizure
Central ocular-motor/positional signs	Persistent positional nystagmus; vertical or direction-changing nystagmus; nystagmus not suppressed by visual fixation; gaze-evoked nystagmus; skew deviation; internuclear ophthalmoplegia
Headache red flags	Progressively worsening headache; morning headache or headache waking the child from sleep; headache with persistent/recurrent vomiting; occipital headache, especially if recurrent or associated with neurologic signs
Signs of raised intracranial pressure or posterior fossa disease	Papilledema; persistent vomiting; gait ataxia or coordination problems; dysarthria; neck pain or torticollis
Meningeal/systemic red flags	Fever; neck stiffness or meningismus
Infants/younger children	Increasing head circumference; tense fontanelle; developmental regression or loss of previously acquired milestones

Note: This checklist is intended as a practical safety filter rather than a diagnostic algorithm. The presence of one or more of these features should lower the threshold for urgent brain MRI and/or expedited neurologic assessment, particularly when the presentation is atypical for benign peripheral or migraine-related phenotypes.

**Table 2:** Phenotype-oriented ancillary testing in pEVS: practical indications and main caveats.

Clinical Presentation/Phenotype	Most Useful Ancillary Tests	Main Caveats/Limitations
Spontaneous attacks without sufficient migraine features and without auditory symptoms (RVC phenotype)	Usually no ancillary testing beyond clinical assessment; consider audiometry and vestibular testing (vHIT, caloric testing, VEMP) in atypical, persistent, or diagnostically uncertain cases	vHIT, caloric testing, and VEMP may reveal abnormalities, but do not reliably distinguish RVC from pVMC/VMC
Spontaneous attacks with migraine features (VMC/pVMC phenotype)	Audiometry when auditory complaints are reported; consider vestibular testing in selected cases; brain MRI if red flags	vHIT, caloric testing, and VEMP may reveal abnormalities, but do not reliably distinguish pVMC/VMC from RVC
Spontaneous attacks with auditory symptoms	Pure-tone audiometry first-line; consider VEMP and caloric testing; IAC/CPA MRI if asymmetrical hearing loss; temporal bone CT if EVAS or third-window pathology is suspected	Transient auditory symptoms may also occur in VMC/pVMC; ancillary findings must be interpreted in clinical context
Ultrabrief, frequent, stereotyped attacks	Audiometry; ABR; high-resolution MRI of the cerebellopontine angle and internal auditory canals	Neurovascular contact or vascular loops on MRI are not diagnostic in isolation; vestibular function tests are often normal or only mildly abnormal
Ultrabrief attacks with altered awareness or other ictal features	EEG and brain MRI; vestibular testing only as needed to exclude peripheral mimics	
Positional attacks	Positional bedside testing is mandatory (Dix–Hallpike, supine roll test, deep head-hanging); brain MRI when central positional nystagmus is suspected	Video-oculography may be useful in documenting and reviewing positional nystagmus
Sound-/pressure-triggered attacks	Pure-tone audiometry, tympanometry, VEMP, and high-resolution temporal bone CT	Imaging alone does not establish the diagnosis; audiovestibular findings must be concordant with symptoms
Orthostatic-triggered dizziness/vertigo and autonomic symptoms	Orthostatic heart rate and blood pressure measurements; ECG when indicated; tilt testing in selected cases	Orthostatic abnormalities may coexist with vestibular migraine or other causes of episodic vertigo/dizziness; findings should be interpreted in the full clinical context
Episodic vertigo with prominent ataxia or other transient neurologic signs	Brain MRI; genetic testing when clinical suspicion is moderate to high; vestibular testing as needed	Vestibular findings are not specific; neurologic and ocular-motor examination often contribute more than vestibular testing
Episodic dizziness associated with anxiety/panic attacks	Diagnosis is usually clinical; consider symptom diaries, orthostatic assessment, and targeted testing only to exclude alternative diagnoses	Ancillary testing is used mainly to exclude alternative vestibular, orthostatic, neurologic, cardiovascular, or metabolic causes; normal results do not by themselves establish PPPD, which is chronic rather than episodic

Abbreviations: ABR, auditory brainstem response; CT, computed tomography; ECG, electrocardiogram; EEG, electroencephalography; EVAS, enlarged vestibular aqueduct syndrome; IAC/CPA, internal auditory canal/cerebellopontine angle; MRI, magnetic resonance imaging; PPPD, persistent postural-perceptual dizziness; pVMC, probable vestibular migraine of childhood; RVC, recurrent vertigo of childhood; VEMP, vestibular evoked myogenic potentials; vHIT, video head impulse test; VMC, vestibular migraine of childhood.

**Table 3:** Diagnostic criteria for recurrent vertigo of childhood (RVC), vestibular migraine of childhood (VMC), and probable vestibular migraine of childhood (pVMC).

Clinical Entity	Diagnostic Criteria
RVC	A. At least three episodes with vestibular symptoms of moderate or severe intensity, lasting between 1 min and 72 h B. None of the criteria B and C for Vestibular Migraine of Childhood C. Age < 18 years D. Not better accounted for by another headache disorder, vestibular disorder, or other condition
VMC	A. At least five episodes with vestibular symptoms of moderate or severe intensity, lasting between five minutes and 72 h B. A current or past history of migraine with or without aura C. At least half of episodes are associated with at least one of the following three migraine features: 1. Headache with at least two of the following four characteristics: (a) One-sided location (b) Pulsating quality (c) Moderate or severe pain intensity (d) Aggravation by routine physical activity 2. Photophobia and phonophobia 3. Visual aura D. Age < 18 years E. Not better accounted for by another headache disorder, vestibular disorder, or other condition
pVMC	A. At least three episodes with vestibular symptoms of moderate or severe intensity, lasting between five minutes and 72 h B. Only one of the criteria B and C for Vestibular Migraine of Childhood C. Age < 18 years D. Not better accounted for by another headache disorder, vestibular disorder, or other condition

RVC: Recurrent vertigo of childhood; VMC: Vestibular Migraine of Childhood; pVMC: Probable vestibular migraine of childhood. Note: Adapted from van de Berg et al. [7].

**Table 4:** Diagnostic criteria for vestibular paroxysmia (VP) and probable vestibular paroxysmia (pVP).

Clinical Entity	Diagnostic Criteria
VP	A. At least ten attacks of spontaneous spinning or non-spinning vertigo B. Duration less than 1 min. C. Stereotyped phenomenology in a particular patient D. Response to a treatment with carbamazepine/oxcarbazepine E. Not better accounted for by another diagnosis.
pVP	A. At least five attacks of spinning or non-spinning vertigo B. Duration less than 5 min C. Spontaneous occurrence or provoked by certain head movements D. Stereotyped phenomenology in a particular patient E. Not better accounted for by another diagnosis.

VP: Vestibular paroxysmia; pVP: Probable vestibular paroxysmia. Note: Adapted from Strupp et al. [26].

**Table 5:** Differentiating BPPV from central positional vertigo/nystagmus in children: bedside discriminators.

	BPPV	CPV/CPN
Latency	Typically, 1–5 s; shorter with rapid positional transitions and in some horizontal-canal phenotypes.	Often 0–5 s; latency alone is not decisive.
Duration of positional nystagmus	Usually <60 s; may be longer in cupulolithiasis.	Often persistent (>60 s) or sustained while the provoking position is maintained
Direction/canal congruence	Canal-congruent (plausible for a semicircular canal plane).	Canal-incongruent vector, or pure vertical/torsional patterns not attributable to a canal plane.
Within-trial spontaneous decay (damping)	Typically damps/decays spontaneously (classically crescendo–decrescendo) while the provoking position is maintained.	May be persistent or show atypical evolution (including direction reversal while holding position).
Fatigability on repetition	Often present in posterior/anterior canal BPPV; less typical in many horizontal-canal presentations.	Often poor/absent in central disorders (variable).
Associated central ocular-motor/neurologic signs	Absent by definition (or clearly separable).	Common (e.g., gaze-evoked nystagmus, saccadic pursuit, dysmetric saccades, ataxia).
Response to appropriate repositioning maneuvers	Typically improves/resolves; immediate disappearance supports diagnosis.	Often refractory to liberatory maneuvers; lack of response should prompt reconsideration.

**Table 6:** Diagnostic criteria for superior canal dehiscence syndrome (SCDS).

Clinical Entity	Diagnostic Criteria
SCDS	A. At least one symptom consistent with the presence of a third mobile window in the inner ear: 1. Bone-conduction hyperacusis 2. Sound-induced vertigo and/or oscillopsia time-locked to the stimulus 3. Pressure-induced vertigo and/or oscillopsia time-locked to the stimulus 4. Pulsatile tinnitus
	B. At least one sign or diagnostic test indicating a third mobile window in the inner ear: 1. Nystagmus characteristic of excitation or inhibition of the affected superior semicircular canal evoked by sound or by changes in middle ear pressure or intracranial pressure 2. Low-frequency negative bone-conduction thresholds on pure-tone audiometry 3. Enhanced VEMP responses (low cervical VEMP thresholds or high ocular VEMP amplitudes)
	C. High-resolution temporal bone CT imaging with multiplanar reconstruction demonstrating dehiscence of the superior semicircular canal
	D. Not better accounted for by another vestibular disease or disorder

Note: Adapted from Ward et al. [43].

**Table 7:** Diagnostic criteria for hemodynamic orthostatic dizziness/vertigo (HODV).

Category	Criteria
HODV—definite	A. ≥5 episodes of dizziness, unsteadiness, or vertigo triggered by arising or during upright posture, relieved by sitting/lying. B. OH, POTS, or syncope documented on standing or head-up tilt. C. Not better accounted for by another disorder.
HODV—probable	A. Same as definite. B. ≥1 associated symptom: weakness/tiredness; difficulty thinking/concentrating; blurred vision; tachycardia/palpitations. C. Not better accounted for by another disorder.

OH = sustained BP fall ≥20/10 mmHg within 3 min of standing/tilt; POTS in adolescents (12–19 years) = orthostatic symptoms + sustained HR increment ≥40 bpm within 10 min without OH. Note: Adapted from Kim et al. [49].

**Table 8:** Diagnostic criteria for motion sickness (MS), visually induced motion sickness (VIMS), motion sickness disorder (MSD), and VIMS disorder (VIMSD). MS is diagnosed when the sickness-inducing stimulus is physical motion of the person; VIMS is diagnosed when the stimulus is visual motion. An acute episode of MS/VIMS is sickness induced by physical motion/visual motion that meets Criteria A through D. Motion sickness disorder (MSD) is diagnosed when the sickness-inducing stimulus is physical motion; visually induced motion sickness disorder (VIMSD) is diagnosed when the stimulus is visual motion. MSD or VIMSD is diagnosed when Criteria A through E are met.

Clinical Entity	Diagnostic Criteria
MS/VIMS	A. Physical motion of the person or visual motion elicits sign(s) and/or symptom(s) in at least one of the following categories, experienced at greater-than-minimal severity: 1. Nausea and/or gastrointestinal disturbance 2. Thermoregulatory disruption 3. Alterations in arousal 4. Dizziness and/or vertigo 5. Headache and/or ocular strain B. Sign(s) and/or symptom(s) appear during motion and build as exposure is prolonged C. Sign(s) and/or symptom(s) eventually stop after cessation of motion D. Sign(s) and/or symptom(s) are not better accounted for by another disease or disorder
MSD/VIMSD	A. At least five episodes of motion sickness/VIMS triggered by the same or similar motion stimuli B. Sign(s) and/or symptom(s) are reliably triggered by the same or similar motion stimuli C. Sign(s) and/or symptom(s) severity does (do) not significantly decrease after repeated exposure to the same or similar motion stimuli D. Sign(s) and/or symptom(s) lead to one or more of the following behavioral or emotional responses a. Activity modification to reduce sickness sign(s)/symptom(s) b. Avoidance of the motion stimulus that triggers sickness c. Aversive anticipatory emotions prior to exposure to the motion stimulus E. Sign(s) and/or symptom(s) are not better accounted for by another disease or disorder

MS: Motion sickness; VIMS: visually induced motion sickness; MSD: motion sickness disorder; VIMSD: VIMS disorder. Note: Adapted from Cha et al. [52].

**Table 9:** Suggested diagnostic criteria for probable episodic ataxia in children and adolescents. If all criteria (A–E) are fulfilled, the diagnosis of “probable episodic ataxia” can be made, and genetic testing should be initiated. A positive genetic finding confirms the diagnosis of episodic ataxia.

Clinical Entity	Suggested Diagnostic Criteria
Probable EA	A. At least three episodes with vertigo, dizziness, and/or ataxia symptoms lasting between 1 min and 72 h
	B. At least half of episodes are associated with at least two of the following features: 1. Speech disturbances 2. Limb ataxia 3. Walking difficulties/having to lie down 4. Double vision/oscillopsia 5. Pallor/weakness
	C. At least one of the following clinical findings in the attack-free interval: 1. Nystagmus (e.g., provocation nystagmus, gaze-holding nystagmus, downbeat or upbeat nystagmus, rebound nystagmus) 2. Impaired smooth pursuit 3. Impaired fixation suppression 4. Impaired optokinetic nystagmus 5. Myokymia or myotonia
	D. Age < 18 years
	E. Not better accounted for by another vestibular or metabolic condition. *

EA: episodic ataxia. Note: Adapted from Filippopulos et al. [55]. * Comorbidity with epilepsy and/or vestibular migraine does not rule out episodic ataxia.

## Data Availability

No new data were created or analyzed in this study.

## Abbreviations

The following abbreviations are used in this manuscript:

ABR	auditory brainstem response
BPPV	benign paroxysmal positional vertigo
BPV	benign paroxysmal vertigo
BPVC	benign paroxysmal vertigo of childhood
CPN	central positional nystagmus
CPV	central positional vertigo
CT	computed tomography
cVEMP	cervical vestibular evoked myogenic potentials
DFNB4	deafness, autosomal recessive 4
EA	episodic ataxia
EA1	episodic ataxia type 1
EA2	episodic ataxia type 2
ECG	electrocardiogram
EEG	electroencephalography
EVA	enlarged vestibular aqueduct
EVAS	enlarged vestibular aqueduct syndrome
EVD	epileptic vertigo/dizziness
HODV	hemodynamic orthostatic dizziness/vertigo
IAC/CPA	internal auditory canal/cerebellopontine angle
ICHD	International Classification of Headache Disorders
ICVD	International Classification of Vestibular Disorders
IHS	International Headache Society
MD	Meniere disease
MRI	magnetic resonance imaging
MS	motion sickness
MSD	motion sickness disorder
OH	orthostatic hypotension
oVEMP	ocular vestibular evoked myogenic potentials
pEVS	pediatric episodic vestibular syndrome
POTS	postural orthostatic tachycardia syndrome
PPPD	persistent postural-perceptual dizziness
pVMC	probable vestibular migraine of childhood
pVP	probable vestibular paroxysmia
RVC	recurrent vertigo of childhood
SCDS	superior canal dehiscence syndrome
SLC26A4	solute carrier family 26 member 4
VEMP	vestibular evoked myogenic potentials
vHIT	video head impulse test
VIMS	visually induced motion sickness
VIMSD	visually induced motion sickness disorder
VMC	vestibular migraine of childhood
VP	vestibular paroxysmia
